# EHPnet: European Chemicals Agency

**Published:** 2008-03

**Authors:** Erin E. Dooley

On 1 June 2007, Europe’s new legislation for managing chemicals, REACH (Regulation, Evaluation, Authorisation and Restriction of Chemicals), entered into force. Under the auspices of REACH, the European Chemicals Agency (ECHA) was set up. ECHA’s scope involves implementing and managing the technical, scientific, and administrative work of REACH as well as working to ensure consistency among the European Union (EU) countries with regard to the legislation. The Helsinki-based agency has established a website at **http://echa.europa.eu/** to provide a central online source for news and information about its work.

The ECHA website has links to information for industry, policy makers, and the general public about the scope of REACH. The REACH page on the website takes visitors to the REACH Navigator, an interactive tool that allows companies to learn more about their responsibilities regarding REACH compliance. Companies find out what they need to do by answering a series of questions posed by the Navigator. The Frequently Asked Questions section addresses general topics as well as questions about specific classes of chemicals. The Guidance section of the REACH page covers the technical aspects of the legislation. Included here are documents explaining the various REACH processes, including registration, data sharing, classification of chemicals, and preparation of chemical safety reports.

The Software Tools page of the ECHA website provides information on REACH-IT and IUCLID 5. REACH-IT allows companies to set up webpages through which they can submit their chemical registration information. Personnel from ECHA and EU member states can use these pages to review this information. REACH-IT also provides a means for companies that manufacture the same chemicals to contact one another, whereas members of the public can use it to find out information on the types of chemicals that are produced in the EU. IUCLID 5 is the International Uniform Chemical Information Database, which companies can use to store and report information on the properties of chemicals. IUCLID 5 uses data-gathering templates developed by the Organisation for Economic Co-operation and Development.

